# Supplementary material for: Patterns of Intron Gain and Loss in Fungi
Source: PLoS Biol. 2004 Nov 30;2(12):e422. doi: 10.1371/journal.pbio.0020422 (PMC532390; doi:10.1371/journal.pbio.0020422)
Supplement: Table S1 — Also available at http://genes.mit.edu/NielsenEtAl/. (4.3 MB ZIP). [file pbio.0020422.st001.zip › NielsenEtAl/html/1311.html]

AN1286.1.NCU04035.1.MG00590.1.FG05344.1


```
 CLUSTAL W (1.82) Multiple Sequence Alignments - Introns Inserted


Sequence 1: NCU04035.1	1028 aa
Sequence 2: FG05344.1	1046 aa
Sequence 3: MG00590.1	1073 aa
Sequence 4: AN1286.1	985 aa
Alignment Length: 1113 aa
Number Identitical Residues: 447 aa
Alignment Score (without introns) 24320


MG00590.1 	-----------MHIIKPS-------WLSHS---G1EQKDFEVYSCHISPDGSRLATAGGD
NCU04035.1	-----------MHLIKPS-------WLSHS---G1EQKDFEVYSCHVSPDGKRLATAGGD
FG05344.1 	-----------MHIIKPS-------WLSHC---G~EQKDFEVYSCHVSPDGKRLATAGGD
AN1286.1  	MDILYRPMLLSIYVCRASRTVLANIFVSFSGFLG~ERKAFEVYSCDVSPDGSRLVTAAGD
          	 .    .   :::: :.* :  :. ::*...  * *:* ******.:****.**.**.**

MG00590.1 	~GHVRVWSTEAIYNADNESFRKPRQLCHMSHHLGTIHSVRFSPNGRYLASGADDKIICIY
NCU04035.1	~GHVRVWSVEAIFNSHDRNYTKPRQLCHMSHHLGTIHSVRFSPNGRYLASGADDKIICIY
FG05344.1 	~GHVRVWSTESIYNANTPDYNKPRQLCHMSHHLGTIHSVRFSPNGRYLASGADDKIICVY
AN1286.1  	1GYVRIWSTEAICGAEDAN--KPKQLASMSNHSGTIHTVRFSPNGKYLASGADDKIVCIY
          	 *:**:**.*:* .:.  .  **:**. **:* ****:*******:**********:*:*

MG00590.1 	QLDSNPPSHTATFG1TNEPPPI~ENWKTYKRLVGHENDVQDIA~WSYDSSILVSVGLDSK
NCU04035.1	HLDSNPP--SHTS-~----TFV1ENWKTYKRLVGHDNDVQDLA~WSPDNSLLVSVGLDSK
FG05344.1 	HLDKNPP--AATFG~TNEPPPI~ENWKTYKRLIGHDNDVQDLA~WSYDSSILVSVGLDSK
AN1286.1  	TLDANPP-------~-------~-------------SHAASFG1WSCDSSILVSVGLDSK
          	 ** ***                             ... .:. ** *.*:*********

MG00590.1 	VVVWSGHTFEKLKTLAVHQSHVKGITFDPANKFFATASDDRTIKIFRFTPPAPNATQHDM
NCU04035.1	IVVWSGHTFEKLKTLAVHQSHVKGITFDPANKFFATASDDRTIKIFRYTAPAPNATQHDM
FG05344.1 	VVVWSGHTFEKLKTLPAHQSHVKGITFDPANKFFATASDDRTIKIFRFTSPAPNATQHDM
AN1286.1  	VVVWSGHTFEKLKTLSVHQSHVKGITFDPANKYFATASDDRTVRIFRFTSPAPNSTAHDQ
          	:**************..***************:*********::***:*.****:* ** 

MG00590.1 	VNNFVLETTINTPFKSSPLTTYFRRCSWSPDGNHIAAANAVNGPVSSVAIIERGSWLSEI
NCU04035.1	VNNFILETSISVPFKHSPLTTYFRRCSWSPDGNHIAAANAVNGPVSSIAIIERTGWDSEI
FG05344.1 	VNNFVLETTISSPFKSSPLTTYFRRCSWSPDGNHIAAANAVNGPVSSVAIIERTRWDSEI
AN1286.1  	MNNFVLEQTITAPFQNSPLTAYFRRCSWSPDGLHIAAANAVNGPVSSVAIINRGGWDGDI
          	:***:** :*. **: ****:*********** **************:***:*  * .:*

MG00590.1 	NLIGHEGPTEVTQFSPRLFHTTDP-ALA~NGNSNGSSNLVTVIASAGQDKTLSIWNTNTS
NCU04035.1	NLIGHEAPTEVCMFSPRLFYTQKPDENS~NANGAASPGLVTVIASAGQDKTLTIWNTNTS
FG05344.1 	NLIGHEAPTEVCMFSPRLFHTSKPDPSV~DDK---SPSLVTVIASAGQDKTLSIWNTNTS
AN1286.1  	NLIGHEAP------------------VE0DSHDHVAQAPVTVIACAGGDKSLSVWITSNP
          	******.*                     : :.  :   *****.** **:*::* *...

MG00590.1 	RPLVILQDLASKSIADLSWTPDGQVLFVCSLDGGIYALKFEEGELGWVATAEENDKALQK
NCU04035.1	RPVLIVQDIASKSVSDLAWTPDGQTVFAASLDGGVIAAQFETGELGWVAKSEENDKALQK
FG05344.1 	RPVVILQDLAGKSVSDLAWTPDGQTLFASSLDGSIVVAKFSEGELGWVAQPEENDKALQK
AN1286.1  	RPIVVAQELAAKSISDLAWSPDGSCLYATALDGTILAVRFEDGDLGYAMELEENEKSLTK
          	**::: *::*.**::**:*:***. ::. :*** : . :*. *:**:.   ***:*:* *

MG00590.1 	YSSSKKGAGTAEDIESLNLENQSKAGELRGAENRMGALMGDALVS--ETSGASPAAATPA
NCU04035.1	YGGSRKGMGTAEDVDGLHLENHSKEKELRGAESRMGALMGD--------PGPAQKETATT
FG05344.1 	YGASRKGMGIAEDVDGLMLENQSKAGESRAVESRMGALMGD--------LPDSTKESTPV
AN1286.1  	FGTNRKGAGIAETTDGLLLEEKSKAGELKGVEGRMGALMGDGHATGDAVANGKPTPLPST
          	:. .:** * **  :.* **::**  * :..*.********.  :..          ...

MG00590.1 	TNGVKAANGDVNGDSTTNNGEKDKDKDKEKEKEKEKDKDKDKDKDASNEGDQAEESEAKK
NCU04035.1	TNGVKPAG-----KAADTNGTTTPAPPEKPE----------------------EESADKT
FG05344.1 	TNGTKTTAP---TSKPATNGQSEAEKDKEPEKEQEKE---------------PEEAADKT
AN1286.1  	SNGVTPAAP-----SPSTDAQKSQPNGTAAPSAS------------------ETEKPDPY
          	:**...:        . .:. .         . .                    *     

MG00590.1 	AERIAALKSRVTITKDGKKRVAPMLVSSSGTGTSSLPQSHLVGKSS-TKAAQADAPQAIL
NCU04035.1	AERIAELKSRVTITKDGKKRVAPLLVSSSGTGLSSLPQAQLVGASS-TKPVQNDNPQTIL
FG05344.1 	AQRVKELKSRVTVGKDGKKRVAPLLVSSSGTGTSSLPQTQLVGTTSNNKAAQNDAPQTTL
AN1286.1  	QAKLERLKQRPTYTKDGKKRIAPLLVSGAGGAESSLPQARLMASVS--SQVKADTPQSIV
          	  ::  **.* *  ******:**:***.:* . *****::*:.  *  . .: * **: :

MG00590.1 	DLTQPFDGLPKGGLASMLLGNKRKAAVLEEDYE---EDTTTKKPHNGPVPVLVNGVEGTE
NCU04035.1	DLSKPYDGLPRGGIAAMLLGNKRKAVIVEDEEE---EEPSAKRSATGPVPIVVNGVEGLE
FG05344.1 	DLAKPFDGLPKGGIAAMLLGNKRRLNLGEAEEE---EEPVSKRAATGPTTIVTNGHDSVE
AN1286.1  	DLSKPFDGLPKGGLATLLFGNKRKLAQLEDEEDGHTEKRVALASQNGATPILTSAPEGLL
          	**::*:****:**:*::*:****:    * : :. :*.  :  . .*...::... :.  

MG00590.1 	PAQLKEPADGVVPTPEVLRPAVLSSSITYSQVRLGVPKVRSHILRPLERGVLQDE--TTL
NCU04035.1	PAPLSAPAHGLVPTPEFLRPAVISPNIAYSQVRLAVPKIRSHILRPLERGVLQEE--TSL
FG05344.1 	PAALVPVQHGVVPTPEFLRPAVMNPSIAFAQVRLAVPKIRSHVLRPLDRGILQGE--SSL
AN1286.1  	PAQPQDPPTGQQPTPEFIRPAVVNPCMSVSQLRLAVPKVRTHIVRAIDSAGKPTEPPSTS
          	**       *  ****.:****:.. :: :*:**.***:*:*::*.:: .    *..:: 

MG00590.1 	EDATKVPENIIFEARNP--------VASRDPSHVTATKRGLLLWHDFLPKAVMLLTGNKN
NCU04035.1	EDATKVPENIVLEAKNP--------AHIREPARITATKRGALLWQDFLPRAIILVAGSKH
FG05344.1 	EDASKTPENIILEAKND--------PNPRDPSHVLVSKRGALIWEEWLPRAIILVTATKQ
AN1286.1  	GESNKSRVDVVFEARNPSGASLTGRAADREPVRLTLFRGEQPLWQDFLPRTVLLVTGNQN
          	 ::.*   ::::**:* :.:: :.    *:* ::   :    :*.::**::::*::..::

MG00590.1 	FWAAACEDGTLYVWSPAGRRLLSAVVLSSQPVILESRGYWLLCITAVGLVHVYNVKTFTS
NCU04035.1	FWAAACEDGTLHTWSPAGRRLMNAIMLESQPVILEARDSWLLCVTAVGLCHVYNIKTMSS
FG05344.1 	FWAVACEDGSIHTWTPAGRRLLAPIILESQPVILEARGHWLLCITAVGLAHVWDLKTQSS
AN1286.1  	MWSAACEDGSVYIWTPAGRRLVSALVLEAQPVILECNGPWILSISAVGMCYVWNVEHLSS
          	:*:.*****::: *:******: .::*.:******... *:*.::***: :*::::  :*

MG00590.1 	PFPPVSLAPILDIAIHTLSPHGPTPGLGVTSAHLNSEGMVVVTLSNGDGFLYSPTMYSWQ
NCU04035.1	PHPPVSLAPILDVAMTSLSPHGATPAPGVTSAHLNSSGVIVVTLSNGDGFYYSTSLYAWQ
FG05344.1 	PSPPVSLGPILDIATTSLNQHSATPGPGVTSAHLNSTGHIIVTLTNGDGYYYAREMFTWQ
AN1286.1  	PHPPVSLQPVLDAAIHTLGAH-PSAAPSITNARINSEGRIIIALSNGEGYAYSPSLYTWQ
          	* ***** *:** *  :*. * .:.. .:*.*::** * ::::*:**:*: *:  :::**

MG00590.1 	RLSETWWAVGSQYWNSNDSSISALQTTAVGPNEAPAPKSQDG-VSVSAGIIPFLERHTTN
NCU04035.1	RLSESWWAVGSQYWNSNDSSVSALQTTAVGPVS--GAKNGEGETNVSAGIIPYLERHTTT
FG05344.1 	RLSEAWWAVGSQYWNSNDSSISALQSTAVGPASKEGKDKESTKATVSSGIIPFLERHTTN
AN1286.1  	RLSEAWWAVGSQYWNSTEAPVGNLQSASN-----TQQKDKDARAAVSAGIIPFLERNTTN
          	****:***********.::.:. **:::         .. .  . **:****:***:**.

MG00590.1 	EFLLRGRAYTLQRLIKTLLSREGFEGFESSVSIAHLENRIAGALQLGSKDEFRLYLFMYA
NCU04035.1	EFLLKGRAYTLQRLVKALLARDGFENFESSVSIAHLENRIAGAFALGAREEFRLYLFMYA
FG05344.1 	EFLLKGRAYGLQRIIKMVVQRKEAENLESSVSIAHLETRIAGALQLGAREEFRLYLFTYA
AN1286.1  	ETLLRGRAYFLQRLIKTLLSREGYESFESSVSIAHLENRLAGALSLGAKEEFRLYLSMYA
          	* **:**** ***::* :: *.  *.:**********.*:***: **:::******  **

MG00590.1 	KRIGAEGLRGKVEELLNSLVGGVLQRRVDGKEEAEGWGWYGK-GELCGWDRRELLKGVVM
NCU04035.1	KRIGAENQKTKVEELLNSLIGGVLR------DADDGEGWFSKQDKLCGWDRKDLLQGVVL
FG05344.1 	KRLGAEGARVKVEELLNSLLGGILEENEEEEDEDDGHGWFSKEGDLCGWDRKELLKDVVM
AN1286.1  	KRIGAEGLRGKVEELLK-------------------------------------------
          	**:***. : ******:                                           

MG00590.1 	ILG1KFRELQRLTVQYARLLGLTADGDDDDDDDNAMSIEQ
NCU04035.1	ILG1KFRDLHRLTVQYARILDMTLGDEEKETTDEGMEVED
FG05344.1 	ILG1KYRELQRLTLQYAKVLDISLEDGP--VDVEQMDVEA
AN1286.1  	--G1KHRDLQRVTVPYAKLLGVVDNESD---ANDAMEL--
          	  * *.*:*:*:*: **::*.:           : *.:
```
